# Effects of Aquatic Exercise on Sleep Quality in Patients with Chronic Diseases: A Meta-Analysis

**DOI:** 10.3390/healthcare14050661

**Published:** 2026-03-05

**Authors:** Shuzhang Zhou, Ming Fang, Billy Chun-Lung So, Hei Wa So, Paul H. Lee, Siushing Man

**Affiliations:** 1 School of Design, South China University of Technology, Guangzhou 510006, China; szzhou@scut.edu.cn (S.Z.); 202521054884@mail.scut.edu.cn (M.F.); ssman6@scut.edu.cn (S.M.); 2 Department of Biomedical Engineering, Hong Kong Polytechnic University, Hong Kong, China; 3 School of Chinese Medicine, Hong Kong Baptist University, Hong Kong, China; 20223897@life.hkbu.edu.hk; 4 Southampton Clinical Trials Unit, University of Southampton, Southampton SO17 1BJ, UK; paul.h.lee@southampton.ac.uk

**Keywords:** aquatic exercise, sleep quality, chronic diseases, meta-analysis

## Abstract

**Background/Objectives:** This study systematically synthesized the evidence on the effectiveness of aquatic exercise (AE)-based interventions for improving sleep quality in patients with chronic diseases and identified key moderating factors. **Methods:** A meta-analysis of 11 randomized controlled trials sourced from Google Scholar, PubMed, Web of Science, Embase, Cochrane Library, and Scopus (published between 2016 and 2025) was conducted. Sleep quality was assessed using subjective tools (e.g., PSQI). **Results:** While AE-based interventions showed potential for enhancing nighttime sleep quality (standard mean difference = 0.825, *p* < 0.001), high statistical heterogeneity (*I*^2^ = 93.41%) was observed. Given this variance, the analysis prioritized the clinical outcomes of specific patient populations over the pooled effect size. Preliminary evidence suggests significant improvements were confirmed in populations with post-COVID syndrome (*p* < 0.001), Parkinson’s disease (*p* = 0.002), and chronic back pain (*p* = 0.008). Conversely, no significant benefits were observed in fibromyalgia (*p* = 0.191), ankylosing spondylitis (*p* = 0.737), or type 2 diabetes (*p* = 0.836). Moderator analysis further indicated that the mode of AE might influence outcomes, with recreational aquatic therapy and deep-water running suggesting superior efficacy compared to resistance training. **Conclusions**: AE-based interventions were suggested as an effective intervention for improving sleep quality. The observed benefits likely stem from the synergistic effects of physical exercise and the unique physiological properties of the aquatic environment, such as buoyancy and hydrostatic pressure. However, the field relies heavily on subjective questionnaires and lacks physiological mechanism studies. These findings provide a preliminary evidence-based framework for clinicians to develop targeted AE-based interventions for chronic disease patients.

## 1. Introduction

Chronic diseases, particularly cardiovascular diseases, cancers, diabetes, and chronic respiratory diseases, have become the leading causes of disability and productivity loss worldwide [1]. Chronic diseases collectively account for over 80% of premature deaths globally (those occurring before age 70) each year. Chronic diseases also inflict annual economic losses exceeding $2 trillion and severely deplete millions of healthy life years [2]. Patients with chronic diseases often face persistent physiological symptoms [3,4] and demanding treatment regimens [5]. These conditions are frequently accompanied by complications, among which sleep disorders represent a particularly prevalent and devastating issue [6]. Sleep disorders, such as insomnia, sleep fragmentation, and non-restorative sleep, significantly diminish chronic disease patients’ sleep quality [7,8]. Poor sleep quality intensifies fatigue among patients with chronic disease, contributes to low mood, and impairs cognitive function [9]. Therefore, developing effective and accessible interventions to enhance the sleep quality of patients with chronic diseases is crucial for enhancing their holistic health and overall well-being.

Clinical practice currently uses various interventions to improve the sleep quality of patients with chronic diseases, mainly pharmacological and non-pharmacological treatments [10,11]. Pharmacological treatments, such as sleeping pills, offer quick symptom relief [12,13], but they often have drawbacks, including daytime drowsiness and the risk of dependency [14,15]. In addition, pharmacological treatments do not address the root causes of poor sleep quality. Given these drawbacks, non-pharmacological approaches are often preferred. For instance, cognitive-behavioral therapy for insomnia is highly effective in improving sleep quality in patients with chronic diseases [16,17]. However, the therapy’s accessibility can be limited and demands high patient adherence and long-term commitment, making consistent implementation challenging for some patients [18,19]. Other lifestyle interventions, such as maintaining a regular sleep schedule and avoiding caffeine and alcohol, are crucial for improving patients’ sleep quality [20]. However, the efficacy of lifestyle interventions can be limited, often proving insufficient for patients with moderate to severe sleep disturbances [21,22]. Some chronic disease patients also face complex physiological constraints or socioeconomic barriers [23,24], making it hard to fully adhere to these interventions. Consequently, the medical community continues to actively explore safe, effective, and sustainable non-pharmacological interventions for enhancing chronic disease patients’ sleep quality [25,26,27].

Land-based exercise (LE) is generally recognized as a valuable non-pharmacological strategy for managing sleep quality in patients with chronic diseases [28,29]. Previous evidence has suggested its potential benefits in promoting sleep continuity and reducing sleep latency. However, LE interventions for many chronic disease patients can present remarkable drawbacks [30,31]. For instance, individuals with arthritis or musculoskeletal conditions might struggle with prolonged or high-intensity land-based activities due to pain or discomfort caused by gravitational load [32,33]. Patients with cardiovascular disease may require stringent exercise monitoring to prevent overexertion [34,35]. These limitations frequently prevent patients from consistently engaging in LE, thereby compromising the intervention’s effectiveness [36,37]. In this context, aquatic exercise (AE), defined as physical activity performed in water (such as water walking, water aerobics, and swimming), has gained increasing attention for its unique advantages [30]. AE is particularly suitable for the chronic disease patient population. Water’s buoyancy effectively reduces joint load, significantly lowering the risk of exercise-related injuries [38].These effects suggest that AE may be a suitable alternative for chronic disease patients with musculoskeletal conditions, arthritis [39,40], obesity [41,42], or mobility limitations [43]. This is primarily because AE substantially mitigates the discomfort and risks often associated with LE [44]. Moreover, the resistance provided by water facilitates a comprehensive workout that may enhance cardiovascular function and muscle strength. These physiological benefits contribute to improved physical fitness while potentially minimizing the mechanical stress imposed on the body [45].

Existing research has shown that AE can positively affect sleep quality through various physiological and psychological mechanisms, thereby enhancing the well-being of patients with chronic diseases. Physiologically, AE may promote melatonin secretion, regulate the sleep–wake cycle, and improve sleep structure [46,47]. Psychologically, the physical and mental relaxation from AE and the warm water environment can relieve anxiety, stress, and depression, common causes of sleep disorders in these patients [48]. Moreover, water’s pain-relieving effects can reduce sleep interruptions caused by physical discomfort and improve sleep quality [49]. Many studies have explored AE’s impact on specific chronic conditions. For diabetic patients, regular AE improved sleep efficiency and total sleep time [50]. Rheumatoid arthritis patients had less pain and fewer sleep interruptions after AE training [51,52]. For obese or cardiovascular disease patients, AE improved sleep quality indicators [53,54]. AE also benefited those with fibromyalgia [55,56] and chronic low back pain [44,57]. Collectively, these studies provided a solid foundation for the idea that AE can improve sleep quality and, by extension, enhance the well-being of patients with chronic diseases.

Previous literature reviews have examined the diverse applications of AE in therapeutic settings, contributing to a deep understanding of this area. Wei, et al. [58] summarized the current state of research on AE therapy for musculoskeletal disorders, highlighting publication trends, influential authors, and evolving research themes. The study identified a shift towards quality-of-life outcomes but did not conduct a quantitative synthesis to evaluate the clinical efficacy of these interventions on sleep quality. Bravo, et al. [59] reviewed 14 studies to evaluate the impact of aquatic therapy on sleep quality and other symptoms in patients with fibromyalgia. The review’s findings highlighted the potential of aquatic therapy to improve self-reported sleep quality and reduce pain in this specific clinical population. Similarly, Santamaría et al. [46] reviewed 13 research articles to examine the effectiveness of aquatic therapy compared to land-based therapy. Their analysis focused on quality of life, balance, and sleep quality among patients with Parkinson’s disease, with results suggesting potential benefits of aquatic interventions in these areas. However, existing research presents several key limitations that have obscured a clear understanding of the overall effectiveness of AE-based interventions in improving sleep quality among patients with chronic diseases. First, a definitive conclusion regarding the effectiveness of AE-based interventions for improving sleep quality across the diverse spectrum of chronic diseases has not been firmly established. While individual studies suggest benefits, the overall therapeutic value of AE-based interventions has not been systematically quantified. Second, the effectiveness of AE-based interventions in improving sleep quality varied across patient populations. AE-based interventions have demonstrated potential benefits for patients with Parkinson’s disease, chronic back pain, and post-COVID syndrome. However, similar improvements have yet to be consistently established in populations with ankylosing spondylitis, fibromyalgia, or type 2 diabetes. Crucially, no rigorous quantitative meta-analysis has been conducted to synthesize these disparate findings and systematically examine the potential reasons for these inconsistencies.

The present meta-analysis was therefore conducted to address the critical research gaps in a synthesized and quantitative understanding of how AE-based interventions impact patients’ sleep quality across the diverse spectrum of chronic diseases. Its primary aim was to provide a robust and overall estimate of the efficacy of AE-based interventions. Another key aim was to investigate how factors such as patient population, intervention protocol, and patient age contribute to heterogeneity and inconsistent findings in the literature. Ultimately, these findings may offer an evidence-based reference for clinicians and therapists. Such insights could support the design of tailored AE programs, potentially enhancing sleep quality, well-being, and overall life quality for patients with chronic diseases.

## 2. Materials and Methods

### 2.1. Literature Search and Screening

Related studies adopted in this meta-analysis were selected through a literature search and sifting of nine online databases (i.e., Google Scholar, PubMed, Web of Science, Embase, Cochrane Library, Scopus, PEDro, CINAHL, and PsycINFO). These comprehensive database searches were performed and finalized on 20 July 2025. The full database-specific search strategies for each platform and the Preferred Reporting Items for Systematic Reviews and Meta-Analyses (PRISMA) 2020 Checklist are provided in Supplementary Materials S1 and S2, respectively. This rigorous approach ensured the comprehensiveness and reliability of the review, aligning with established systematic review practices in similar technology-enhanced psychological interventions [60]. Formal English publications from 2016 through 2025 were prioritized to reflect contemporary evidence regarding AE and sleep quality. This screening protocol effectively filtered out unpublished works, such as theses and conference abstracts, to ensure a robust review of the last ten years. Notably, full-text preprints were retained provided they met all methodological requirements, as they offer a comprehensive account of evidence that may be undergoing peer review. The inclusion of full-text preprints was considered a strategic measure to capture a broader research landscape. This approach was further justified as a means to mitigate potential publication bias [61]. The identification strategy relied upon the keywords detailed below: (“water-based exercise” OR “aquatic exercise”) AND (“sleep quality” OR “sleep disorder” OR “insomnia” OR “sleep efficiency” OR “sleep efficacy”) AND (“effect” OR “impact”) AND (“randomized controlled trial” OR “randomised study” OR “RCT”). Further screening was based on the following inclusion criteria:

- Considering the feature of this study, the selected literature should evaluate the effectiveness of AE on sleep quality. Therefore, the literature that did not account for the relevant influence of AE was removed.
- The literature inclusion criteria required that studies describe at least one specific procedure for AE application and focus on patients with chronic diseases. Thus, publications failing to provide a comprehensive account of the experimental design and materials were deemed ineligible for inclusion.
- The adoption criteria for this study were strictly limited to adult patients diagnosed with chronic diseases. Therefore, studies focusing on special populations, such as pregnant women, children, elite athletes, or individuals in the acute phase of illness, were excluded from this analysis.
- Studies were required to clearly describe the interventions and control protocols. For the experimental group, interventions involving AE alone or AE combined with LE were included. For the control group, both active controls (e.g., LE) and passive controls (e.g., no exercise [NE] or usual care) were deemed eligible. Publications failing to provide a comprehensive account of these experimental designs were excluded.
- To verify the analytical validity of AE, studies were screened for data completeness, necessitating the explicit presentation of sample size, impact variables, and *p*-values. Articles devoid of these essential quantitative details were subsequently filtered out.

### 2.2. Data Extraction and Coding

All identified records were imported into EndNote 21 for systematic management. Duplicate records were removed using both the automated de-duplication function and subsequent manual verification. Following categorization based on the inclusion criteria, two reviewers independently performed data extraction from all eligible studies. The primary effect sizes quantified the impact of AE-based interventions on sleep quality in patients with chronic diseases. These values were subsequently used to analyze and compare intervention effects across the included studies. Following Cohen [62], effect sizes were derived using mean scores and standard deviations. Extracted data further consisted of author information, year of publication, sample size, and demographic information (e.g., mean age, gender, country or region, and patient population), intervention strategies, control conditions, outcome measurements, intervention duration, frequency, session duration, and water depth. A third party settled any disputes regarding the data.

Moderating variables were coded according to the protocol outlined in Table 1. These included population characteristics, intervention strategies, control conditions, mode of AE, and outcome measurements.

### 2.3. Quality Assessment

To evaluate the risk of bias (RoB), the study employed the Cochrane RoB 2 tool [73]. The study examined five specific domains: randomization, intervention adherence, missing data, outcome assessment, and reporting bias. Independent assessments were conducted by two researchers, who graded each domain as “low,” “some concerns,” or “high.” A “high” rating indicated the presence of significant bias, whereas “some concerns” reflected a lack of clarity in the reported data. The overall trial quality was based on the worst-scoring domain: if any domain was rated as “high,” the entire study was classified as high risk. Similarly, the presence of “some concerns” in any domain (without high risk) resulted in an overall rating of some concerns. Disagreements were arbitrated by a third investigator.

### 2.4. Statistical Analysis

Statistical computations were executed via Comprehensive Meta-Analysis 3.0. To ensure cross-scoring consistency, a sign inversion procedure was applied to subjective sleep measures, specifically the Pittsburgh Sleep Quality Index global score, the Korea Sleep Scale A, and the sleep indicator of the Nottingham Health Profile. Since lower raw scores on these scales represent greater sleep disturbance, their mean values were multiplied by −1, while standard deviations remained unchanged [74]. This transformation aligned all instruments onto a single continuum where higher values consistently signify better sleep quality and a positive intervention effect. The alignment facilitates an intuitive interpretation of the pooled standardized mean difference [75].

To maintain statistical independence, a “one outcome per study” approach was adopted [75]. For studies reporting multiple sleep outcomes, the primary endpoint or the most validated scale (PSQI) was prioritized to provide a single, representative point estimate for the primary analysis. For multi-arm trials, the sample size of the shared control group was divided to avoid unit-of-analysis errors [76]. Subsequently, a random-effects model was applied to assess the effect size distribution. This approach was widely adopted in meta-analyses within occupational health [77] and technology assessment [78] to account for anticipated heterogeneity across studies. Study heterogeneity was assessed using *Q* and *I*^2^ statistics.

Furthermore, moderator analyses were performed to investigate potential sources of heterogeneity across the included studies. The study specifically examined eight potential moderators based on the coding protocol: mode of AE, patient population, mean age, intervention strategies, intervention duration, control conditions, frequency, and session duration. The statistical significance of the moderators was assessed using the *Q*-test. To address the concern regarding evidence stability, a sensitivity analysis was conducted by excluding the preprints from the meta-analysis. For publication bias, both Begg’s and Egger’s tests were considered [79,80,81]. Due to its superior efficacy and sensitivity in small samples [82], Egger’s test was primarily utilized alongside fail-safe N and funnel plots [83]. The interpretation of Egger’s test and funnel plots was approached cautiously because bias detection power is inherently limited with a modest number of studies [84].

## 3. Results

### 3.1. Results of the Screening

Conducted in strict adherence to the PRISMA guidelines [61], the review process initially identified 3163 records from the electronic databases. The study protocol was prospectively registered on the PROSPERO international prospective register of systematic reviews (ID: CRD420261307422).

**Figure 1:**
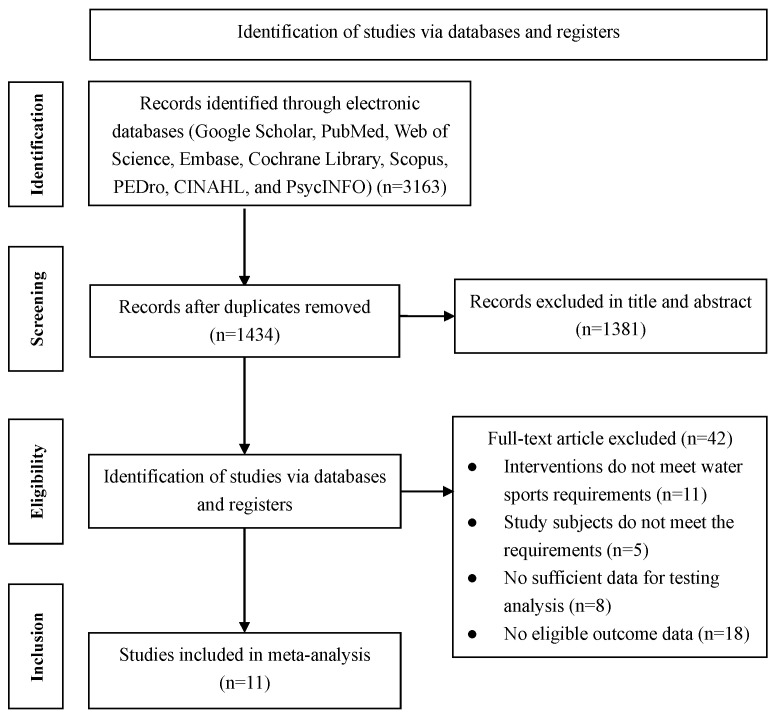
PRISMA flowchart of the study screening process.

The application of specific keywords and eligibility criteria ultimately narrowed this selection down to 11 qualifying studies. The detailed progression of this screening is depicted in Figure 1.

### 3.2. Coding Details of Included Studies

Key characteristics of the included studies were categorized to facilitate detailed analysis. Intervention strategies were stratified into two types: AE and AE + LE. Control conditions were divided into LE and NE. The male-to-female ratio (M/F) was classified into four categories: M/F = 0, 0 < M/F < 1, M/F > 1, and M/F = 1. The outcome measures were categorized into three types: Pittsburgh Sleep Quality Index global total score, Korea Sleep Scale A, and sleep indicator of Nottingham Health Profile. Geographically, the trials originated from diverse regions, including Brazil, China, France, Korea, Spain, and Turkey. The patient population was classified into six groups: ankylosing spondylitis, chronic back pain, fibromyalgia, Parkinson’s disease, post-COVID syndrome, and type 2 diabetes. Regarding the specific content, the mode of AE comprised four forms: aquatic aerobic exercise, aquatic resistance training, deep-water running, and recreational aquatic therapy. Exercise dosage was documented across three dimensions: intervention duration (3–15 weeks), session duration (typically 45–60 min), and training frequency (2–6 sessions per week). Finally, water depth was documented based on specific measurements (e.g., 120 cm, 130–150 cm) or marked as “not available (NA)” when details were unavailable. Table 2 presents the detailed coding for each study.”

Table 3 summarizes the experimental designs of 11 studies investigating AE interventions, providing a detailed comparison between intervention protocols and control treatments. Key parameters such as water temperature, exercise intensity monitoring, and specific aquatic techniques are documented.

### 3.3. Risk of Bias Assessment

**Figure 2:**
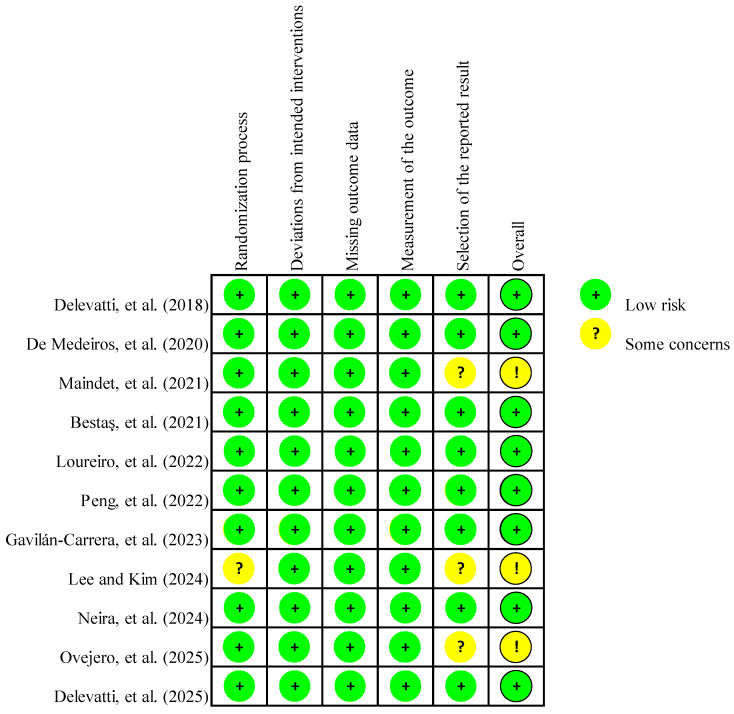
Risk of bias summary for the included studies: Delevatti, et al. [85], De Medeiros, et al. [86], Maindet, et al. [87], Bestaş, et al. [88], Loureiro, et al. [89], Peng, et al. [90], Gavilán-Carrera, et al. [91], Lee and Kim [92], Neira, et al. [93], Ovejero, et al. [94], Delevatti, et al. [50]. The symbols represent: (+) Low risk; (?) Some concerns; (!) Overall judgment of some concerns.

Figure 2 summarizes the risk of bias of included studies, indicating no high-risk studies, eight studies with low risk, and three studies with some concerns across all domains. Notably, this methodological rigor was supported by a robust randomization process, with ten studies reporting valid random sequence generation. However, the study by Lee and Kim [92] was judged to have a risk of randomization. Additionally, all studies reported a low risk regarding deviations from intended interventions and missing outcome data. Consequently, there was a low risk of performance and attrition bias across these specific domains. Similarly, the outcome measurements were judged to be of low risk across all studies, resulting in an overall low risk. The risk of reporting bias was judged as unclear in three studies, primarily due to selective outcome reporting. No additional sources of bias, such as industry funding or conflicts of interest, were identified.

### 3.4. Overall Effect Size

**Figure 3:**
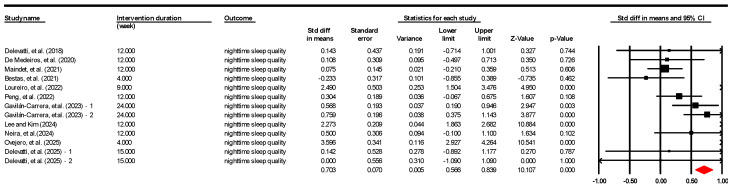
Forest plot grouped by measurements using a random-effects analysis for the included studies: Delevatti, et al. [85], De Medeiros, et al. [86], Maindet, et al. [87], Bestaş, et al. [88], Loureiro, et al. [89], Peng, et al. [90], Gavilán-Carrera, et al. [91], Lee and Kim [92], Neira, et al. [93], Ovejero, et al. [94], Delevatti, et al. [50].

Eleven studies contributing 13 effect sizes were examined to assess the impact of AE-based interventions on sleep quality in chronic disease patients (Figure 3). The analysis yielded an overall effect size of 0.825 (95% C.I. = 0.266–1.384; *p* < 0.001) for sleep quality improvement (Table 4).

For the heterogeneity tests, the results of the random effects meta-analysis are shown in Table 4. Higgins and Thompson [95] claimed that heterogeneity exists when *I*^2^ exceeds 75%. In Table 4, *I*^2^ is 93.41%, indicating considerable statistical and clinical heterogeneity across the records. Given this variance, the subsequent moderator analysis was prioritized to explain the efficacy across specific patient populations.

### 3.5. Moderator Analysis

Given the heterogeneity observed among the included records in Section 3.4, it was necessary to investigate the underlying sources of variance. In meta-analyses of exercise interventions, particularly within clinical populations, high heterogeneity is relatively common and expected [96]. Inherent differences in participant demographics, clinical conditions, and the diverse parameters of exercise protocols across studies often stem from this variance [97]. To systematically explore potential sources of variation, moderator analysis was conducted to provide a treatment of this high heterogeneity [98]. The mode of AE, patient population, intervention strategies and control conditions were treated as categorical variables, while mean age, intervention duration, frequency, and session duration were analyzed as continuous variables. These analyses were performed using random-effects and maximum-likelihood methods. According to Peng and Chan [99], the coefficient represents the marginal effect (standardized mean difference). This value is derived by calculating the difference between the reference category and the focal category. Table 5 presents the results of the single-covariate meta-regression for the entire dataset. The mode of AE (*Q* = 9.12; *p* = 0.0278) and patient population (*Q* = 30.31; *p* < 0.01) significantly influenced the effect size. The corresponding *R*^2^ values for the mode of AE and patient population were 0.34 and 0.73.

The mode of AE played a crucial role in influencing the effect size. Relative to recreational aquatic therapy, shifts to aquatic aerobic exercise, deep-water running, and aquatic resistance training reduced the effect sizes by 1.21, 1.65 and 1.94, respectively. No statistically significant difference was observed between aquatic aerobic exercise and deep-water running. Furthermore, the patient population was observed to be the main significant moderator of the effect size. Subgroup analysis showed significant improvements in Parkinson’s disease (*p* = 0.002), chronic back pain (*p* = 0.008), and post-COVID syndrome (*p* < 0.001). Conversely, no significant improvements were observed in fibromyalgia (*p* = 0.191), ankylosing spondylitis (*p* = 0.737), and type 2 diabetes (*p* = 0.836).

When the patient population shifted from the reference group (Parkinson’s disease) to ankylosing spondylitis, fibromyalgia, and type 2 diabetes, the effect sizes significantly decreased by 2.72, 2.09, and 2.39, respectively. Crucially, the analysis revealed no statistically significant difference in effect size for the chronic back pain and post-COVID syndrome populations compared to Parkinson’s disease, indicating comparable efficacy. In contrast, no statistically significant associations were identified for the remaining covariates (*p* > 0.05). Specifically, the intervention strategies and control conditions did not significantly predict treatment outcomes. Similarly, intervention duration, frequency, and mean age showed no significant association with effect sizes.

### 3.6. Test and Adjustment for Publication Bias

Publication bias represents the tendency to report and publish studies with statistically significant results, rather than those with invalid or results [100]. In this study, publication bias was assessed using the Begg and Mazumdar rank correlation test [101] and Egger’s regression intercept test [83]. The results showed no significant evidence of publication bias in either analysis. Begg’s test yielded a Kendall’s tau of 0.179 (*p* = 0.393), and similarly, Egger’s test indicated no significant bias (intercept = 1.828, *p* = 0.535). To further evaluate the robustness of the results, Rosenthal’s classic fail-safe N was employed, which estimates the number of unpublished null studies required to nullify the observed effect. The analysis yielded a fail-safe N of 347, a figure much larger than the required 75 (13 × 5 + 10). Therefore, unpublished studies were considered to have no significant effect on the outcomes, showing that publication bias was low.

The trim-and-fill method was employed to estimate the unbiased effect size. No missing points were imputed on the left side, suggesting that the observed overall sleep quality effect sizes were not substantially inflated by publication bias. Consequently, the nighttime sleep quality standardized mean difference remained unchanged at 0.825 (95% C.I. = 0.266–1.384) for the primary outcome. Furthermore, a sensitivity analysis was conducted to evaluate the impact of the preprint on the overall findings. Following the exclusion of the preprint, the overall effect size adjusted from 0.825 to 0.691. Crucially, the statistical significance across all outcomes remained unchanged. This sensitivity check demonstrated that while the preprint influenced the magnitude of the effect, the primary findings and conclusions remain robust.

## 4. Discussion

This study suggested the potential efficacy of AE-based interventions over control conditions in improving sleep quality in patients with chronic diseases. The factors influencing the effectiveness were also identified. The present results corroborated previous findings regarding the comparative benefits of AE-based interventions over control conditions for improving sleep quality in patients with chronic diseases. This convergence of evidence may offer meaningful clinical implications. AE-based interventions could be considered as a valuable non-pharmacological option for sleep improvement, especially for individuals with chronic diseases who may face barriers to LE.

This investigation advanced the current understanding of AE-based interventions in three primary ways. First, it indicated the possible extent and direction of the difference between AE-based interventions and control conditions regarding sleep enhancement in chronic disease populations. Second, notable heterogeneity in the comparison of effectiveness between AE-based interventions and control conditions was observed across the studies included. Third, this study identified several factors that may modulate the effectiveness of AE-based interventions, with mode of AE and patient population being the specific contributors identified.

### 4.1. Effectiveness of AE-Based Interventions on Sleep Quality

This meta-analysis indicated a trend favoring AE-based interventions over control conditions for improving sleep quality in chronic disease populations, a conclusion based on the overall effect sizes derived from eleven comparative studies. Notably, AE-based interventions showed a potential for outperforming control conditions in nighttime sleep quality measurements (*p* < 0.001). Nighttime sleep quality was primarily assessed using instruments such as the Pittsburgh Sleep Quality Index, the Nottingham Health Profile, and the Korea Sleep Scale A questionnaire. These tools collectively measured patients’ overall perception of sleep duration, efficiency, and disruption. These findings suggested that AE-based interventions may better enhance patients’ nighttime sleep quality, especially among those with chronic conditions.

Nighttime sleep quality encompasses physiological indicators such as sleep continuity and the frequency of nocturnal awakenings. It also includes the subjective experience of waking up feeling energetic, mentally alert, and physically refreshed [102]. The unique physical environment of AE-based interventions may address these aspects, creating distinct therapeutic conditions that could improve the patients’ sleep experience. Moreover, the current lack of objective sleep data limited the ability to conduct robust analyses of heterogeneity and publication bias. Future research is strongly recommended to apply objective measurement tools such as actigraphy [103] or polysomnography [104] to clarify the effects on sleep quality of AE-based interventions. While subjective questionnaires are valuable for capturing patient perceptions, they can introduce measurement variability. This variability may obscure the true sources of between-study heterogeneity, a common challenge in behavioral intervention research [105,106]. In contrast, objective indicators such as sleep efficiency and wake after sleep onset provide highly consistent and reliable data. These indicators are essential for a precise estimation of the pooled effect size and a trustworthy assessment of publication bias [107,108]. Furthermore, objective data are crucial for elucidating the mechanisms underlying AE by clarifying its primary effects on sleep onset latency, sleep architecture, and arousal frequency [106,109].

### 4.2. Moderating Variables

#### 4.2.1. Mode of AE

The mode of AE-based interventions was associated with variations in the overall effect size for sleep quality in patients with chronic diseases (*p* = 0.0278). Specifically, when using recreational aquatic therapy as the reference, the effect size for aquatic resistance training was significantly smaller. Conversely, when the mode shifted to aquatic aerobic exercise or deep-water running, the effect size difference compared to recreational aquatic therapy did not reach statistical significance. Recreational aquatic therapy, aquatic aerobic exercise, and deep-water running appear to be effective intervention modes for the targeted populations. In contrast, specific muscle training may be comparatively less suitable for improving sleep quality in patients with chronic diseases.

The substantial decrease in benefit observed for aquatic resistance training suggests possible limitations in improving the sleep quality of patients with chronic diseases. First, isolated muscle-strengthening training lacks the necessary cardiopulmonary and nervous system effects for improving sleep quality in patients with chronic diseases [110]. Recreational aquatic therapy (such as continuous dynamic practice) can effectively improve cardiopulmonary function and energy metabolism, thereby promoting relaxation and physical tiredness, which are required for deep sleep. At the same time, recreational aquatic therapy also improves balance, coordination, and proprioception, thereby regulating the autonomic nervous system to enhance sleep comfort and continuity [111]. Moreover, recreational aquatic therapy (such as aquatic group games) is typically aimed at promoting deep relaxation, fostering a positive atmosphere and providing systemic conditioning. These psychological and emotional benefits are likely to significantly improve sleep quality. Furthermore, the repetitive and tedious nature of specific muscle training can lead to low participant motivation and adherence. In contrast, recreational aquatic therapy fosters high compliance through its emphasis on high enjoyment, strong interactivity, and positive psychological gain [112].

Aquatic aerobic exercise and deep-water running showed no statistically significant difference compared with recreational aquatic therapy, indicating that they have similar overall efficacy in improving sleep quality in patients with chronic diseases. This similarity in efficacy is likely because, while different in form, aquatic aerobic exercise and deep-water running are also whole-body activities. They provide comparable cardiopulmonary and nervous system benefits to those of recreational aquatic therapy, which are essential for improving sleep quality in patients with chronic diseases [113]. Consequently, when designing AE protocols to enhance the sleep quality of patients with chronic diseases, the choice of exercise form could be flexibly determined based on the specific preferences and personal goals of patients with chronic diseases [114]. This flexible approach does not strictly require the adoption of a recreational aquatic therapy.

#### 4.2.2. Patient Population

The overall benefit of AE-based interventions appeared significantly lower in patients with ankylosing spondylitis (*p* = 0.737), fibromyalgia (*p* = 0.191), and type 2 diabetes (*p* = 0.836) compared to the reference group (Parkinson’s disease). However, AE demonstrated no significant benefit over the reference group in patients with chronic back pain (*p* = 0.008) or post-COVID syndrome (*p* < 0.001). The success of AE-based interventions may not be universal and could depend on the alignment between the therapy’s effects and the disease’s core pathology. Specifically, the intervention needs to target the underlying mechanisms that disrupt sleep in each specific condition. This potentially explains why efficacy varies across different patient populations.

For patients with Parkinson’s disease (*p* = 0.002), chronic back pain, and post-COVID syndrome, AE-based interventions can alleviate core symptoms that directly disrupt sleep, including movement impairment, pain, and functional decline [115]. In patients with Parkinson’s disease, AE-based interventions help alleviate sleep disturbances by targeting muscle stiffness. The buoyancy of water supports the body weight while the warmth promotes muscle relaxation. This physical relief allows for greater ease of movement in bed. It lessens the bodily discomfort from being unable to change sleeping positions during the night, which typically leads to frequent awakening [116]. In chronic back pain, the mechanism is similar but focuses on the spine. AE-based interventions take the pressure off the spinal discs. This relaxes tight back muscles and stops the pain from keeping patients awake [117]. For post-COVID syndrome, the water pressure helps the body calm down. AE-based interventions shift the nervous system away from stress. This makes it easier to fall asleep by reducing the physical alertness often seen in these patients [118].

Conversely, for patients with ankylosing spondylitis, type 2 diabetes, and fibromyalgia, AE-based interventions may not adequately address the deep or complex pathological mechanisms causing sleep disturbances [115]. In fibromyalgia, the brain processes pain too intensely. AE-based interventions help relax the muscles. However, they cannot stop the brain from making the pain feel worse [119]. Similarly, in ankylosing spondylitis, sleep is disturbed by inflammation throughout the body. AE-based interventions may provide temporary comfort. Yet, they do not control the internal inflammation as well as strong medicine [120]. Finally, the results in type 2 diabetes were limited because the sleep causes are different. Sleep problems here often come from blood sugar changes or breathing pauses. AE-based interventions may not directly fix these issues. They are better used as an extra help rather than the main solution [121].

#### 4.2.3. Mean Age

Meta-regression under the random-effects model revealed no significant association between mean age and effect size. This lack of significance is likely attributable to aggregation bias, given that the included trials recruited participants across widely spanning age ranges (e.g., 18–65, 30–75, or over 30 years). The reliance on study-level means to represent such heterogeneous cohorts masked individual-level physiological differences, thereby reducing the model’s sensitivity to detect genuine age-related trends [122,123].

#### 4.2.4. Intervention Strategies

No statistically significant distinction was observed between the intervention strategies based on the random-effects meta-regression. However, this finding implies insufficient statistical power due to severe subgroup imbalance rather than a definitive lack of efficacy. As detailed in the study characteristics, the analysis contrasted only two trials utilizing the combined intervention against nine trials in the AE reference group. This scarcity of data points in the combined group likely contributed to the inflated standard error, thereby potentially masking any additive benefits of the combined modality [123].

#### 4.2.5. Intervention Duration, Frequency, and Session Duration

Meta-regression analyses were conducted to examine the potential dose–response relationship of the AE-based interventions. The results indicated that neither intervention duration nor training frequency showed a statistically significant linear association with effect sizes. Regarding session duration, a quantitative regression was not pursued due to the pronounced homogeneity in the data, as the vast majority of trials utilized a standardized protocol of 45–60 min per session.

Collectively, these findings suggest that the variations in dosage within the included studies were insufficient to drive differential outcomes. The lack of statistical significance likely reflects a ceiling effect or an optimal therapeutic window [124]. The prevailing protocols (typically 12 weeks, 2–3 sessions/week, 50 min/session) appear to be sufficient to induce physiological adaptations conducive to sleep improvement. Consequently, extending the duration beyond 12 weeks or increasing frequency beyond three times weekly may not necessarily guarantee incremental benefits in this specific population [125].

#### 4.2.6. Control Conditions

Moderator analysis revealed no significant difference in effect sizes between studies using LE and those using no exercise NE as control groups (*p* > 0.05). This lack of differentiation suggests that, within the included studies, the therapeutic contrast between LE and NE was insufficient to alter the relative superiority of AE-based interventions.

A primary reason for this may be the suboptimal efficacy of LE interventions in chronic disease populations. Patients might face gravity-induced physical strain and pain during land-based activities, which can lead to lower exercise intensity or reduced adherence compared to aquatic-based interventions. Consequently, the actual physiological gains in the LE group may not have been substantially higher than those in the NE group, especially concerning sensitive outcomes like sleep quality. Unlike LE, the aquatic environment may offer synergistic physiological support through hydrostatic pressure and thermal regulation, which independently promote muscle relaxation and autonomic stability [126]. These water-specific benefits, combined with the intervention, create a therapeutic gap that remains consistent whether compared against an active or passive control. Therefore, the observed “no difference” between control conditions likely reflects the inherent limitations of land-based exercise for these specific populations, further highlighting the unique clinical value of AE-based interventions [88].

### 4.3. Theoretical and Practical Implications

#### 4.3.1. Theoretical Implications

A comprehensive assessment of the eligible studies identified several methodological limitations worth discussing. First, several studies were constrained by small sample sizes, such as those by Delevatti, S, Schuch, Kanitz, Alberton, Marson, Lisboa, Pinho, Bregagnol, Becker and Kruel [85] (2018; n = 21) and Loureiro, Burkot, Oliveira and Barbosa [89] (2022; n = 28). Such small samples may increase the risk of chance findings and reduce statistical power, which may contribute to the between-study heterogeneity observed in this meta-analysis [127]. Limited participant numbers in studies can obscure smaller yet significant effects, leading to imprecise estimates prone to outlier influence and amplifying heterogeneity in meta-analyses. Larger trials are needed to obtain more reliable estimates of treatment effects, reduce heterogeneity, and bolster meta-analysis conclusions.

Second, assessments of sleep quality within the included studies were insufficiently comprehensive, with most studies measuring only nighttime sleep quality and relying heavily on subjective self-reports. This narrow approach fails to fully capture the multidimensional impact of AE-based interventions on the sleep quality of patients with chronic diseases. Future studies should prioritize in-depth and specific assessment strategies to evaluate the effects of AE-based interventions on sleep quality in chronic disease populations. Adopting such approaches would likely enhance the reliability and generalizability of subsequent research findings [128]. This strategy advocates for a dual-approach assessment, combining scales like the PSQI with objective physiological monitoring (e.g., PSG, actigraphy) to obtain multidimensional sleep metrics [129].

Third, the lack of comprehensive research into the mechanisms by which AE-based interventions improve sleep quality in patients with chronic diseases is a notable significant deficiency in the current literature. Aquatic sports may enhance sleep quality in patients with chronic diseases by leveraging the physical properties and psychological mechanisms of the water environment. Physiologically, warm water at 34−36 °C may promote blood vessel dilation, accelerates the drop in core body temperature, and regulate sleep patterns [130]. Hydrostatic pressure enhances parasympathetic nerve activity, thereby reducing cortisol levels. Buoyancy reduces the gravitational load, alleviates muscle spasm, and induces meditative brain waves [126]. Psychologically, the underwater environment reduces sensory input, lowers the activity of the brain’s default network, and combines the blue space effect to alleviate anxiety [131]. The mastery of aquatic skills may boost an individual’s self-efficacy. Simultaneously, team-based interactions foster social connections and could improve mood, a process potentially mediated by the release of prosocial neuropeptides such as oxytocin [132]. Despite promising theoretical frameworks, the specific physiological and psychological mechanisms underlying the effects of AE-based interventions on sleep remain largely unexplored. A comprehensive investigation of these pathways is currently absent from the existing literature. Future research should focus on understanding the physiological and psychological mechanisms behind the efficacy of buoyancy in reducing gravitational load and alleviating muscle spasms rather than merely confirming it.

Fourth, current research significantly overlooked the factor of water depth. Specifically, among the 11 studies analyzed, eight did not provide specific descriptions of water depth. This lack of clarity severely limited the ability to explore differences in the effects of AE-based interventions on sleep across various water depths and made it difficult to accurately assess the moderating role of water depth. A detailed analysis of water depth is crucial because it directly impacts the water’s buoyancy, resistance, and pressure on the human body. These physical factors can further influence exercise intensity, cardiovascular load, and bodily sensations, potentially affecting the physiological responses and final sleep quality of patients with chronic diseases [133]. Therefore, future research should pay close attention to and meticulously document water depth, thereby enabling a comprehensive understanding of the potential benefits of AE-based interventions for the sleep quality of patients with chronic diseases.

#### 4.3.2. Practical Implications

The results of this meta-analysis suggested several direct implications for clinical practice. First, the analysis of specific modalities indicated that recreational aquatic therapy, aquatic aerobic exercise, and deep-water running may be effective in improving sleep quality in patients with chronic diseases. Based on these observations, such modalities could be considered for prioritization in clinical practice. Second, AE-based interventions appeared more effective than control conditions in enhancing sleep quality for individuals with Parkinson’s disease, post-COVID syndrome and chronic back pain. Consequently, AE-based interventions could be considered as complementary therapies for these populations, especially where LE is less tolerated. Third, the analysis did not identify a clear linear dose–response relationship regarding intervention dosage. This finding supports the use of standard, moderate-dosage protocols. For example, sessions of 45–60 min 2–3 times per week may be appropriate. Such protocols could potentially maximize cost-effectiveness and minimize patient fatigue.

### 4.4. Limitations

This review acknowledges several primary limitations. First, the number of eligible studies remains modest (n = 11). Some studies provided incomplete reporting, which precluded precise effect size extraction. Therefore, future research is encouraged to mandate full data transparency. All outcomes should be reported in detail, regardless of statistical significance.

Second, the high statistical heterogeneity (*I*^2^ = 93.41%) reflects the diverse clinical profiles of the included populations. While subgroup analyses were performed, the distinct pathophysiology of each disease may still influence the overall findings.

Third, the inconsistent reporting of water depth in the primary studies resulted in data gaps that could not be filled. Water depth is a critical factor in aquatic exercise, as it directly influences hydrostatic pressure and buoyancy, which may subsequently affect physiological responses and sleep outcomes [133]. This lack of standardized reporting is a notable limitation, as immersion level modulates venous return and autonomic nervous system activity [134]. Therefore, future research should provide detailed measurements of water depth to enable a more robust analysis of its specific impact on sleep outcomes.

Fourth, most included studies compared AE with LE or NE, rather than a control group involving “head-out water immersion without exercise”. Consequently, isolating the effects of physical exertion remains challenging, as outcomes may be partially attributed to the balneotherapy-like benefits (e.g., buoyancy and hydrostatic pressure) inherent to the aquatic environment [133]. These physical factors alone may modulate autonomic nervous system activity, potentially confounding the exercise-specific outcomes. Future research could employ a three-arm trial (AE, passive water immersion, and land-based control) to distinguish the impact of the aquatic environment from that of physical activity on sleep outcomes.

Fifth, while statistical tests suggested low publication bias, it must be noted that these assessments are inherently underpowered when the number of included studies is small [84]. Consequently, the current results regarding publication bias should be interpreted with caution, as the limited sample size may have restricted the ability to detect potential reporting asymmetries.

Finally, this review relies heavily on subjective tools like the PSQI. The lack of objective measurements, such as actigraphy [103] or polysomnography [104], limits the understanding of physiological sleep architecture changes. Future studies should integrate these objective metrics to provide more robust evidence.

## 5. Conclusions

This meta-analysis synthesized 13 specific datasets derived from 11 articles published over the past decade. The standardized mean differences were calculated between AE-based interventions (AE or AE + LE) and control conditions (LE or NE). These values were used to evaluate their comparative efficacy in improving sleep quality for patients with chronic diseases. The results suggested that AE-based interventions may offer advantages over control conditions, with the overall effect size for nighttime sleep quality being 0.825. Mode of AE and patient population appeared to significantly moderate the effectiveness of AE-based interventions in improving sleep quality. Consequently, these findings strongly supported the promotion and inclusion of AE-based interventions in the management of chronic diseases.

However, it should be noted that the reliance on self-reported measures in the included studies may introduce recall bias and affect the precision of the findings. To address the potential discrepancy between subjective perceptions and physiological sleep states, future research should prioritize the inclusion of objective measurements. These implications suggest several key directions. First, non-pharmacological sleep interventions should be more personalized. Second, clinical rehabilitation guidelines should include AE-based interventions. Third, healthcare institutions should implement policies to expand program access. The integration of tools such as actigraphy or polysomnography is strongly recommended in future studies to provide more robust evidence.

## Figures and Tables

**Table 1:** Protocol of coding moderating variables.

Variable	Category	Coding Method
Outcome measurements	Nighttime sleep quality: PSQI, KSSA	PSQI: Pittsburgh Sleep Quality Index global total score. KSSA: Korea Sleep Scale A. NHP: Sleep indicator of Nottingham Health Profile.
M/F	M/F > 1; M/F = 1; 0 < M/F < 1; M/F = 0	M/F > 1: More males than females. M/F = 1: Equal numbers of males and females. 0 < M/F < 1: More females than males. M/F = 0: Only females.
Mode of AE	AAE; ART; DWR; RAT	Aquatic aerobic exercise (AAT): Continuous, rhythmic whole-body movements targeting cardiovascular fitness [63]. Aquatic resistance training (ART): Targeted aquatic resistance training exercises utilizing water drag force or auxiliary equipment [64]. Deep-water running (DWR): Simulated running in deep water using flotation aids, with no foot contact with the pool floor [65]. Recreational aquatic therapy (RAT): Game-based activities, social interaction tasks, or relaxation techniques focusing on enjoyment and psychophysical well-being [66].
Patient population	Ankylosing spondylitis; Chronic back pain; Fibromyalgia; Parkinson’s disease Post-COVID syndrome Type 2 diabetes	Ankylosing spondylitis: A chronic inflammatory rheumatic disease primarily affecting the axial skeleton, characterized by sacroiliitis, spinal stiffness, and progressive structural damage [67]. Chronic back pain: A condition characterized by persistent pain or discomfort in the lumbosacral region lasting for more than 12 weeks, often without a specific identifiable pathology [68]. Fibromyalgia: A chronic disorder characterized by widespread musculoskeletal pain, accompanied by fatigue, sleep, memory, and mood issues, diagnosed based on specific symptom severity scales [69]. Parkinson’s disease: A progressive neurodegenerative disorder primarily affecting the motor system, characterized pathologically by the loss of dopaminergic neurons in the substantia nigra and the presence of Lewy bodies [70]. Post-COVID syndrome: A condition defined by the persistence of symptoms usually 3 months from the onset of COVID-19 that last for at least 2 months and cannot be explained by an alternative diagnosis [71]. Type 2 diabetes: A chronic metabolic condition characterized by insulin resistance and relative insulin deficiency, leading to persistent hyperglycemia and associated systemic complications [72].
Intervention strategies	AE; AE + LE	AE: Aquatic exercise. AE + LE: Combination of aquatic exercise and land-based exercise.
Control conditions	LE; NE	LE: Land-based exercise. NE: No exercise and accept normal treatment.

**Table 2:** Summary of included studies in this meta-analysis.

Article Number	Author (Year)	Sample Size	Intervention Strategies	Control Conditions	Outcome Measurements	Demographic Information				Mode of Aquatic Exercise	Intervention Duration (Week)	Frequency (*n*/Week)	Session Duration (Minute)	Water Depth (cm)
						Mean Age (Years)	Gender (M/F)	Country/ Region	Patient Population					
01	Delevatti, et al. [85]	21	AE	LE	PSQI	56.8	0 < M/F < 1	Brazil	Type 2 diabetes	DWR	12	3	IG: 45 CG: 55	NA
02	De Medeiros et al. [86]	42	AE	LE	PSQI	48.1	M/F = 0	Brazil	Fibromyalgia	AAE	12	2	IG: 40 CG: 50	120
03	Maindet, et al. [87]	188	AE	NE	PSQI	49.8	0 < M/F < 1	France	Fibromyalgia	ART	3	6	IG: within 120 CG: 0	NA
04	Bestaş, et al. [88]	40	AE	LE	PSQI	40.3	M/F > 1	Turkey	Ankylosing spondylitis	ART	4	5	IG: 60 CG: 60	NA
05	Loureiro, et al. [89]	28	AE + LE	LE	PSQI	66.0	M/F > 1	Brazil	Parkinson’s disease	RAT	9	2	IG: 65 CG: 30	120
06	Peng, et al. [90]	113	AE	NE	PSQI	31.0	0 < M/F < 1	China	Chronic back pain	ART	12	2	IG: 60 CG: 60	130–150
07	Gavilán-Carrera, et al. [91]	250	AE	LE/NE	PSQI	50.8	M/F = 0	Spain	Fibromyalgia	ART	24	3	IG: 45–60 CG: 0	120
08	Lee and Kim [92]	152	AE	NE	KSSA	72.1	M/F = 0	Korea	Chronic back pain	AAE	12	2	IG: 60 CG: 60	NA
09	Neira, et al. [93]	38	AE	LE	PSQI	50.0	M/F = 0	Spain	Fibromyalgia	RAT	12	3	IG: 60 CG: 60	120
10	Ovejero, et al. [94]	98	AE	NE	PSQI	48.5	0 < M/F < 1	Spain	Post-COVID syndrome	ART	4	3	IG: 15 CG: 0	NA
11	Delevatti, et al. [50]	44	AE/AE + LE	LE	PSQI	58.1	M/F = 1	Brazil	Type 2 diabetes	AAE	15	3	IG: 60 CG: 60	NA

Abbreviations: AE = aquatic exercise; LE = land-based exercise; NE = No exercise; KSSA = Korea Sleep Scale A; NHP = sleep indicator of Nottingham Health Profile; PSQI = Pittsburgh Sleep Quality Index global total score; AAE = aquatic aerobic exercise; ART = aquatic resistance training; DWR = deep water running; RAT = recreational aquatic therapy.

**Table 3:** Characteristics of intervention groups and control groups.

Article Number	Intervention Details	Control Treatment
01	Deep-water running using a flotation vest, 30 °C, 85–100% *HR_AT_*	Land-based running on athletic track
02	Continuous aquatic aerobic exercise, 32 °C, Borg 12–14	Mat Pilates
03	Supervised pool exercises and high-pressure underwater jets massage	Usual care
04	Muscle strengthening and flexibility in pool	Land-based strengthening
05	Recreational group activities (ball games, hiking) combined with individual passive WATSU relaxation, 34 °C, and land-based therapy	Land-based conventional therapy
06	Therapeutic strength and core stability training, 29–31 °C, Borg 12–14	Physical therapy modalities
07	Water-based resistance exercises using drag equipment, 60–70% *HRmax*	Usual care
08	Aquarobics, 29–30 °C, RPE 11–13	Educational program
09	Group recreational therapeutic exercises, 32–34 °C, Borg CR-10: 3–5	Land-based therapeutic exercise
10	Calisthenics and mobilization in pool, 34 °C	No intervention
11	Two groups: (1) Aerobic circuit training alternating lower/upper limbs, 30 °C, 85–100% *HR_AT_*. (2) Combined training: Identical aerobic circuit training and land-based therapeutic exercise	Sedentary control

Abbreviations: *HR_AT_*: Heart Rate at Anaerobic Threshold; *HRmax*: Maximum Heart Rate; Borg 12–14: Borg Rating of Perceived Exertion 6–20 Scale; RPE: Rating of Perceived Exertion Borg; CR-10: Borg Category-Ratio 0–10 Scale.

**Table 4:** Effectiveness of methods between AE-based interventions and control conditions with a random effects meta-analysis.

Effect Size and 95% Confidence Interval					Test of Null (2-Tailed)		Heterogeneity			
Number of Records	Point Estimate	Standard Error	Variance	95% C.I.	Z-Value	*p*-Value	*Q*-Value	*df*(*Q*)	*p*-Value	*I*^2^ (%)
13	0.825	0.285	0.081	(0.266–1.384)	2.894	0.004	181.987	12	<0.001	93.406

**Table 5:** Results of three independent univariate meta-regressions regarding moderator analysis.

Covariate	Coefficient	Z-Value	2-Sided *p*-Value	Test to Model	*R* ^2^
Mode of AE (reference to recreational aquatic therapy)				*Q* = 9.12; *df* = 3; *p* = 0.0278	
Intercept	2.15	4.30	<0.001		0.34
Aquatic aerobic exercise	−1.21	−1.78	0.075		
Deep-water running	−1.65	−1.54	0.123		
Aquatic resistance training	−1.94	−2.6	0.009		
Patient population (reference to Parkinson’s disease)				*Q* = 30.31; *df* = 5; *p* < 0.0001	
Intercept	2.49	3.25	0.001		0.73
Ankylosing spondylitis	−2.72	−2.69	0.007		
Chronic back pain	−1.21	−1.38	0.169		
Fibromyalgia	−2.09	−2.55	0.011		
Post-COVID syndrome	1.11	1.08	0.278		
Type 2 diabetes	−2.39	−2.70	0.007		

## Data Availability

No new data were created or analyzed in this study.

## Supplementary Materials

The following supporting information can be downloaded at: https://www.mdpi.com/article/10.3390/healthcare14050661/s1. File S1: Full database-specific search strategies for all included platforms; File S2: Preferred Reporting Items for Systematic Reviews and Meta-Analyses (PRISMA) 2020 Checklist.
